# Radiation-induced changes of reactive astrocyte distribution in mice as a late response to partial-brain proton irradiation

**DOI:** 10.2340/1651-226X.2025.44056

**Published:** 2025-07-23

**Authors:** Robin Hegering, Sindi Nexhipi, Theresa Suckert, Johannes Soltwedel, Elke Beyreuther, Mechthild Krause, Antje Dietrich, Armin Lühr

**Affiliations:** a Department of Physics, TU Dortmund University, Dortmund, Germany; b OncoRay – National Center for Radiation Research in Oncology, Faculty of Medicine and University Hospital Carl Gustav Carus, Technische Universität Dresden, Helmholtz-Zentrum Dresden-Rossendorf, Germany; c German Cancer Consortium (DKTK), Partner Site Dresden, and German Cancer Research Center (DKFZ), Heidelberg, Germany; d Helmholtz-Zentrum Dresden-Rossendorf, Institute of Radiooncology – OncoRay, Dresden, Germany; e DFG Cluster of Excellence ‘Physics of Life’, TU Dresden, Dresden, Germany; f Helmholtz-Zentrum Dresden-Rossendorf, Institute of Radiation Physics, Dresden, Germany; g Department of Radiotherapy and Radiation Oncology, Faculty of Medicine and University Hospital Carl Gustav Carus, Technische Universität Dresden, Dresden, Germany; h National Center for Tumor Diseases (NCT), Partner Site Dresden, Germany; i German Cancer Research Center (DKFZ), Heidelberg, Germany; j Faculty of Medicine and University Hospital Carl Gustav Carus, Technische Universität Dresden, Dresden, Germany; k Helmholtz Association / Helmholtz-Zentrum Dresden-Rossendorf (HZDR), Dresden, Germany

**Keywords:** Proton therapy, late radiation effects, mouse brain irradiation, astrocytes, blood–brain barrier, glial fibrillary acidic protein

## Abstract

**Background and purpose:**

After proton therapy of brain tumors, several studies have reported late image changes in follow-up magnetic resonance imaging, which result from blood–brain barrier (BBB) disruption. Astrocytes play a central role in the formation and maintenance of the BBB. To study the late response to partial-brain proton irradiation, preclinical mouse data were utilized to investigate the spatial distribution and dose dependence of reactive astrocytes.

**Material and methods:**

Previously, C57BL/6JRj mice were irradiated with protons targeting the right hippocampal region with single prescription doses of 45–85 Gy. After six months, mice were sacrificed and the excised brains axially cut into 3 μm thick slices and stained for glial fibrillary acidic protein (GFAP) to target astrocytes. Here, a workflow to segment the GFAP-positive area on slice images was established. The fraction of GFAP-positive area (GFAP+ fraction) was evaluated in the high-dose region in the right hemisphere and in the mirrored region in the left hemisphere. Dose distributions were simulated on pre-irradiation cone-beam computed tomography and co-registered to the histological slices.

**Results:**

For all irradiated mice, the GFAP+ fraction in the right hemisphere was significantly increased compared to the left hemisphere and to a sham-irradiated mouse with a highly symmetric GFAP distribution. The GFAP+ fraction in the right hemisphere increased approximately linearly with prescription dose. For comparable doses, the cerebral cortex showed lower GFAP+ fractions than the midbrain.

**Interpretation:**

GFAP upregulation correlated with dose level and distribution. In combination with other markers and timepoints, these findings contribute to a comprehensive understanding of cellular response.

## Introduction

Proton therapy is an increasingly accessible option for highly conformal radiation therapy. The dose of protons increases with increasing depth in tissue and reaches a maximum – the Bragg peak – shortly before the protons stop. Bragg peaks are typically positioned in and adjacent to the target volume, resulting in a substantial reduction in integral dose to healthy tissue. Nevertheless, late radiation-induced side effects were observed months or years after treatment for brain tumor patients as image changes in magnetic resonance imaging (MRI) that could not be explained by the physical dose distribution alone [1–4]. These late-occurring lesions are associated with radiation-induced blood–brain barrier (BBB) disruption or, in some cases, with radiation necrosis [5] that can severely impair the quality of life [6, 7].

Current investigations focus on understanding the development of these lesions and identifying markers for prognostic or therapeutic applications. This requires insights into the tissue changes down to the cellular level with respect to the spatial distribution of induced reactions through experimental in vivo data, as these cannot be gained from human patients. A recently published multimodal dataset involving partial-brain irradiation of mice reproduced the MRI image changes observed in patients [8, 9], which result from local contrast agent leakage due to BBB disruption. Thereby, it resembles clinical relevant symptoms in contrast to most preclinical models that perform whole- or half-brain irradiation [10]. Histological observations revealed gliosis, vessel proliferation and dilation, incomplete necrosis, siderophages, and reduced neuronal cell density [11]. This dataset provides extensive whole-brain histology of several histological markers alongside MRI, cone-beam computed tomography (CBCT) and proton dose distribution [8, 9] to study cell response associated with late radiation-induced side effects.

Astrocytes are the most abundant glial cells and have important roles during the brain’s development, its homeostasis, and response to injury [12]. They are a key component of the BBB, playing an important role in its formation and maintenance [6, 13]. After irradiation, astrocytes become activated and, together with activated microglia, mitigate damage by glial scar formation and immune-cell recruitment [14]. Depending on the severity and location of the injury, the gliosis can be transient (leading to healing) or persistent. The latter leads to continuing glial cell reactivity and disruption of the BBB, and might eventually escalate to neurotoxicity or necrosis [5, 12]. However, regional differences of radiation response are recognized [2, 3], but underlying mechanisms are not yet understood. A characteristic feature of astrocyte reactivity is elevated glial fibrillary acidic protein (GFAP) expression [15], and severity of injury was found to correlate with the quantity of GFAP expression [14, 16].

The aim of this study was to investigate dose dependence and spatial distribution of reactive astrocytes within mouse brains after focused proton beam irradiation by analyzing brain slices stained for GFAP.

## Material and methods

### Animal cohort and slice selection

Images of mouse brain slices originated from a previous study and animal procedures and histological staining protocols are described elsewhere in detail [8, 9, 11] (version 0.3.1). In brief, female mice were irradiated at a high-precision proton beamline, targeting the right hippocampus with a collimated beam (4 mm diameter) at different dose levels with a single fraction. Mice were sacrificed after an observation period of six months or at previously defined endpoints to prevent severe burden. Formalin-fixed paraffin-embedded brains were axially cut (3 μm) with an interval of 100 μm and stained for GFAP and DAPI (4´,6-diamidino-2-phenylindole) to detect astrocytes and nuclei, respectively [8, 17]. Images of the brain slices (resolution 0.65 μm × 0.65 μm) were graded with a quality score between 1 (poor) and 5 (excellent) according to tissue integrity and artifacts, as described earlier [18]. For each mouse, dose simulations on the CBCTs were conducted with the Monte Carlo framework ‘Tool for Particle Simulation’ (TOPAS) [19].

In this study, four mice from the C57BL/6JRj strain were considered from the dataset: one mouse per prescription dose level (45 Gy, 65 Gy, and 85 Gy) and a sham-irradiated animal. For each mouse, three mid-brain GFAP slices were considered, graded with quality scores of 4–5 and containing the isodose line of 80% of the maximum dose.

### Image registration and astrocyte segmentation

**Figure 1:**
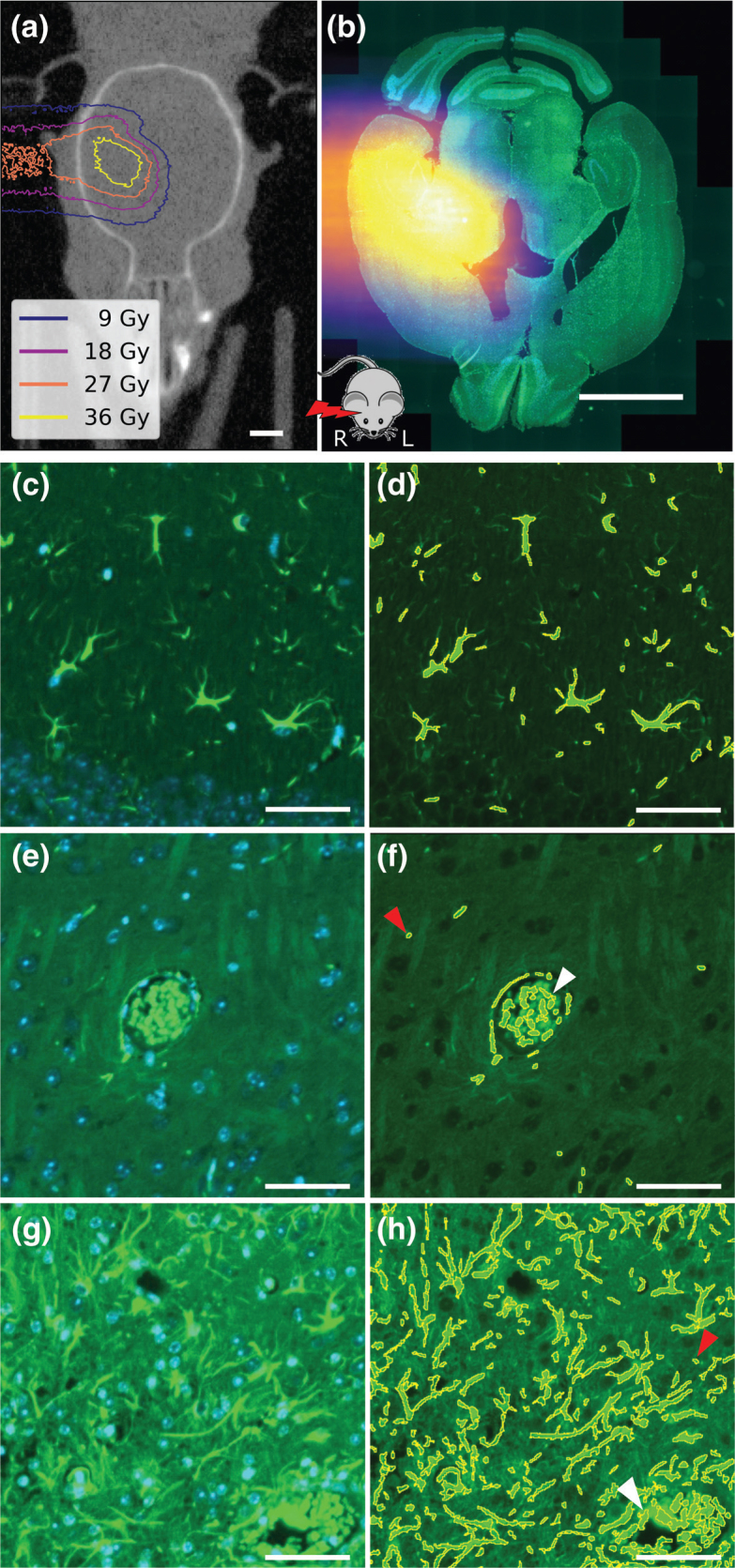
(a) Cone-beam computed tomography image of mouse head with overlaid simulated dose distribution as isodose lines (dose levels corresponding to 20%, 40%, 60%, and 80% of 45 Gy prescription dose). (b) Brain slice image for the same mouse stained with glial fibrillary acidic protein (GFAP, green) and 4´,6-diamidino-2-phenylindole (DAPI, blue) overlaid with the registered proton dose distribution. The mouse sketch on the bottom indicates the relative orientation of proton beam and mouse. (c, e, and g) Representative images of three tiles (250 μm × 250 μm) from different slices stained for GFAP and DAPI. (d, f, and h) GFAP overlaid with segmentation results for the same tiles. (c) and (d) originate from the cortex region of a sham-irradiated mouse. (e) and (f) show a blood vessel aligned with astrocytes in the region of interest (ROI) of another slice from the sham-irradiated mouse. (g) and (h) originate from the right ROI of an 85 Gy irradiated mouse. White arrows indicate false-positive staining of erythrocytes in the vessels. Red arrows indicate potential false-positive autofluorescent segments. Scale bars: (a–b) 2 mm; (c–h) 50 μm.

The simulated dose distribution was spatially correlated and upscaled into the coordinate system of each individual brain slice [20] (Figure 1b) using the Slice2Volume registration algorithm [8], realizing contour-based image transformation between the CBCT and the histological slices.

For GFAP segmentation, microscopy image preprocessing was done using Fiji (version 2.16.0) [21]. The local background of the GFAP slices was subtracted with the rolling ball function [22] with a radius of 3.25 μm. Afterward, image intensities were transformed to an 8-bit scale and intensity thresholding was performed with the Otsu algorithm [23] to segment the GFAP signal (Figure 1c–h). Segments with areas below 7 μm² or above 1,000 μm² were excluded, but erythrocytes and autofluorescence were not. Within tiles measuring 250 μm × 250 μm, the GFAP-positive area fraction (GFAP+ fraction) was determined by dividing the summed-up area of all segmented parts by the tile area.

### Statistical analysis

**Figure 2:**
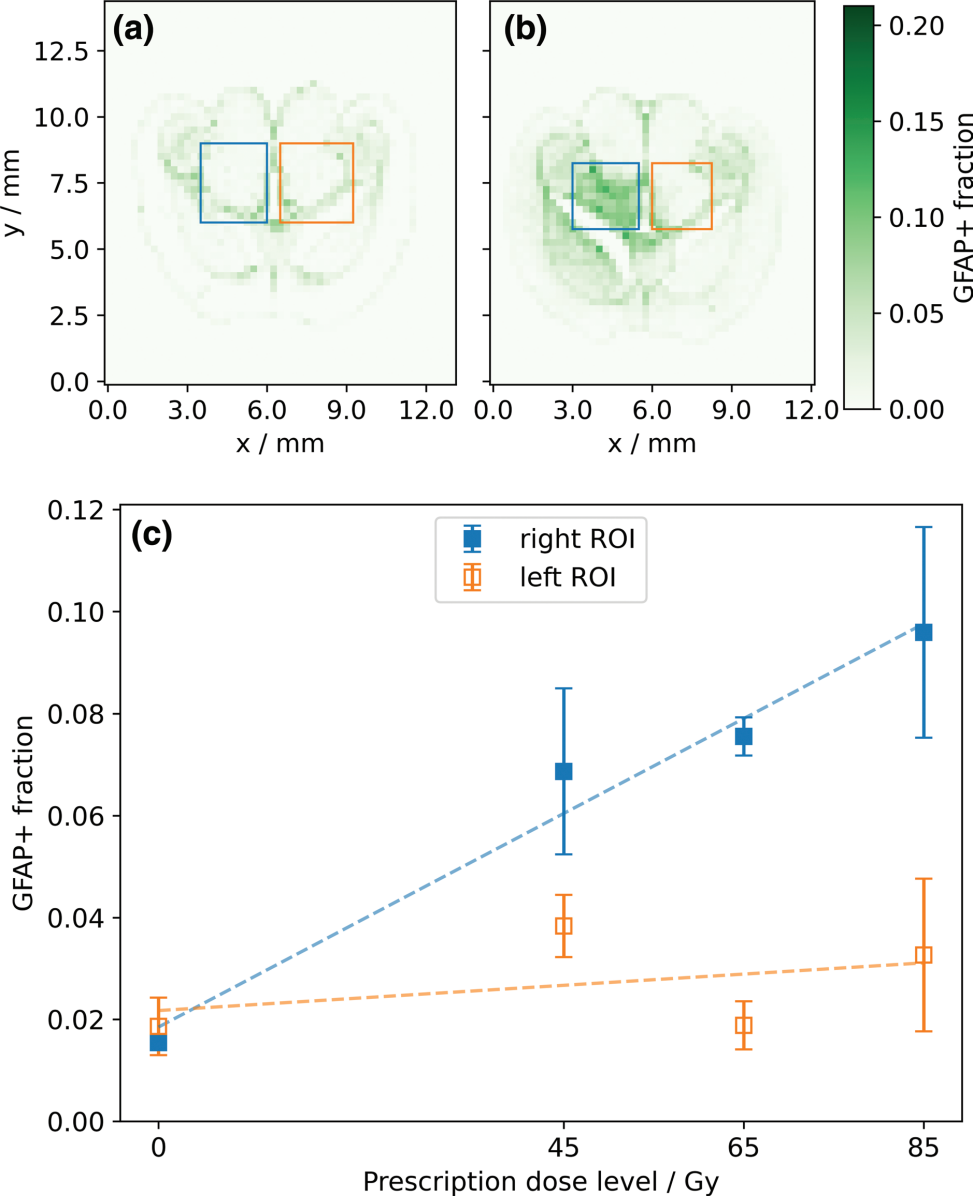
Exemplary histograms representing the fraction of glial fibrillary acidic protein (GFAP)-positive area per 250 μm × 250 μm tile (color bar) with delineated regions of interest (ROIs) in the right (blue) and left (orange) hemisphere: (a) sham-irradiated and (b) 65 Gy mouse. (c) Mean GFAP-positive area fraction (GFAP+ fraction) of the right and left ROIs for different levels of prescription dose, averaged over three slices per dose level with one standard deviation. Dashed lines show results from linear regression.

To analyze the response of the GFAP signal to radiation, a rectangular region of interest (ROI) of approximately 2.4 mm × 2.4 mm was defined in both the right and left hemispheres of the brain slices, centered along the beam axis (Figure 2a). In the right (irradiated) hemisphere, the ROI contained the high-dose region in the midbrain (thalamus, midbrain, and hippocampus), excluding the cortex toward the lateral and the third ventricle toward the medial side. This ROI was mirrored in the left hemisphere, respecting the anatomical landmarks. Within each ROI, the mean GFAP+ fraction over all tiles was calculated and correlated against the prescription dose. The increase with dose was determined using linear least-squares regression and tested for a difference from 0 at a significance level of 0.05 using a Wald test with t-distribution using scipy.stats.linregress [24].

**Figure 3:**
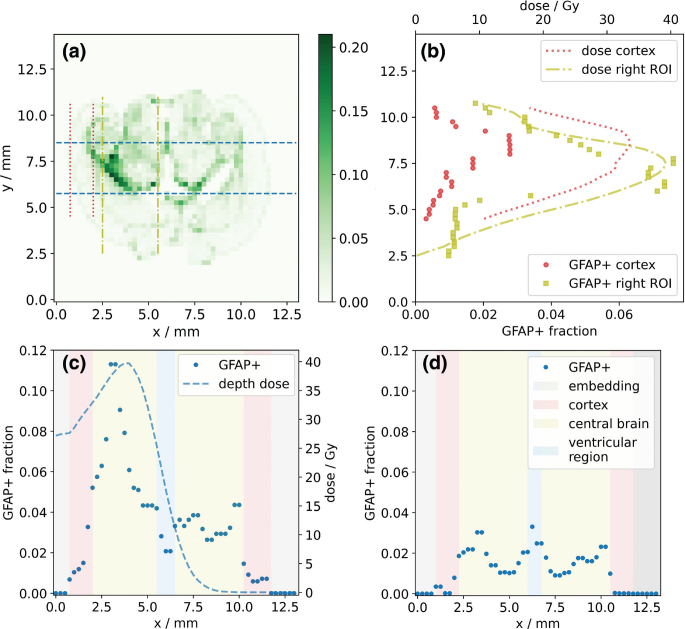
(a) Representative histogram showing the fraction of glial fibrillary acidic protein (GFAP)-positive area per 250 μm × 250 μm tile (color bar) for a 45 Gy mouse brain slice. Dashed lines indicate integration intervals for the dose and GFAP profile along the beam axis (blue, dashed) and in two areas transverse to the beam axis, which represent the cerebral cortex (red, dotted) and the central brain region (yellow, dash-dotted). (b) Transverse dose and GFAP response profiles in cortex (red) and central brain region (yellow). Depth-dose and GFAP response profiles (blue, dashed) for (c) the 45 Gy and (d) a sham-irradiated mouse brain slice. Background colors indicate anatomical regions. Note that the x-axis of (c) and the y-axis of (b) correspond to the respective x- and y-axes of (a).

The spatial variation of the GFAP+ fraction and the simulated dose distribution were compared along the beam direction. For this purpose, the mean values were calculated for all tiles perpendicular to the beam direction within the width of the right ROI (i.e., between the blue dashed lines in Figure 3a).

To investigate how anatomical regions influence the dose response transverse to the beam direction, the tile values along the beam direction were averaged separately within the cerebral cortex (red dotted lines) and in the depth of the target region (central brain, yellow dash-dotted lines).

## Results

GFAP-positive areas were segmented on nine irradiated and three sham-irradiated slices. Figures 1c, e, and g show three representative tiles with the corresponding segmentation result added in Figures d, f, and h, respectively. Overall, the method segmented the GFAP-positive area with high quality, which means that the segmentation depicts astrocytes. In both the irradiated and sham-irradiated examples, small GFAP-positive objects were segmented. These might include cross-cut astrocyte processes but cannot be distinguished from autofluorescent structures of the same size. Furthermore, erythrocytes were segmented due to their high autofluorescent signal in the green channel (Figure 1f, and h, arrows).

For the sham-irradiated mouse, the distribution of the GFAP+ fraction was symmetrical between both hemispheres (Figure 2a). In contrast, all slices from irradiated mice showed an increase in GFAP+ fraction in the ROI in the right (irradiated) hemisphere relative to that in the left hemisphere (Figure 2b). Comparing all slices, the GFAP+ fraction in the right ROIs increased significantly (*p* < 0.05) and approximately linearly with prescription dose (Figure 2c). The contralateral ROIs in the left hemispheres showed substantially lower GFAP+ fractions for all irradiated slices, and higher prescription doses did not result in a significant (*p* > 0.5) increase in the GFAP+ fraction.

In general, GFAP expression and its increase with dose were not evenly distributed in the brain slices, with a marked increase in GFAP at some anatomical boundaries (Figures 2a and b, 3a). Along the anterior-posterior axis (perpendicular to the proton beam direction), the GFAP response agreed, in general, well with the shape of the dose profile. However, in the cerebral cortex region, a lower increase of the GFAP+ fraction with dose was observed compared to more central brain regions (Figure 3b). For irradiated slices, analysis of the dose and corresponding GFAP response showed that the maxima of these two curves occurred at similar depths along the proton beam direction in the brain (Figure 3c). In the non-irradiated hemispheres, GFAP+ fractions were mostly comparable to those of the sham-irradiated mouse (Figure 3d). However, some elevation of the GFAP+ fraction was observed in proximity to the third ventricle and where the dose gradient at the end of the proton beam was located. For the sham-irradiated slices, the GFAP profile showed a symmetrical shape along the hypothetical beam direction in both brain hemispheres (Figure 3d), with GFAP+ fractions in the cerebral cortex being substantially smaller than those in central brain regions.

## Discussion and conclusion

This study investigated the impact of focused proton dose on the spatial distribution of GFAP+ fraction in mice that developed late radiation-induced image changes on MRI due to BBB disruption. Analyses of whole-brain histology revealed a significant increase in GFAP expression on the irradiated hemisphere that followed the spatial distribution of the proton dose in the brain and scaled linearly with prescription dose, while no significant increase in GFAP expression was observed on the contralateral hemisphere. In addition to dose, the respective anatomical region had an impact on the observed level of GFAP+ fraction. In particular, the cerebral cortex showed a lower GFAP expression without irradiation and a less pronounced increase with dose.

The known correlation of the quantity of GFAP expression in reactive astrocytes with the severity of injury [14] together with the observed linear scaling of GFAP expression with prescription dose in the high dose region suggests that the extent of radiation-induced late damage also scales with dose. This is consistent with the severity of the BBB disruption observed for the analyzed mice, which also increased markedly with the prescription dose [11]. Additionally, the increased GFAP signal in the irradiated regions aligns well with previous findings on hypertrophy of astrocytes following injury [15, 16, 25]. Moreover, dose dependence and regional differences in radiation response were also observed for activated microglia that were investigated on the same open dataset [18].

Earlier work using a single X-ray fraction showed that higher doses (20–45 Gy) led to increased astrogliosis, which persisted for 1 year, whereas lower doses did not affect the number of GFAP-expressing astrocytes [26]. Wilson et al. reported a significant increase in the number of GFAP+ astrocytes at 24 and 48 h after a single dose of 20 Gy localized cranial X-ray radiation. Notably, in the same mice, they observed a correlation between radiation-induced increases in BBB permeability and the induction of astrogliosis in this acute phase [27]. However, a study investigating the impact of high dose rates (FLASH) of electron irradiation reported lower GFAP response compared to conventional rates [28], as used here.

In line with the current work, cortical astrocytes in healthy mouse brains have a lower GFAP content than hippocampal astrocytes [14]. Others, however, saw a significant increase in astrocyte number, which was relatively homogeneous throughout white matter, 150 days after single-fraction whole-brain X-ray dose of more than 30 Gy, not restricted to specific areas or clusters [29].

A major limitation of this exploratory study is the small animal cohort, with only one mouse per dose group, and thus, the results have to be validated using a bigger sample size. Moreover, the evaluated regions only approximately corresponded to the respective anatomical regions. For example, the regions considered to be cortex also included the anatomical boundary between cortex and hippocampus, resulting in a local peak of GFAP+ fraction near the boundary. In a future study, brain region atlas-based registration could enable improved delineation of anatomy [30] and thus, the evaluation of more complex anatomical structures, such as the periventricular region, which has been shown to exhibit increased radiosensitivity and microglia activity [2, 18]. Spatial uncertainties of the simulated dose relative to the histological slices were estimated to be 1.5 mm and 0.5 mm along and across the beam direction, respectively, with lateral positioning validated through proton radiography in treatment position [17]. These were mainly caused by uncertainties in the dose simulation on pre-treatment CBCT and the subsequent registration with histological slices. However, the observed spatial correspondence of dose and GFAP+ profiles indicates that spatial registration generally worked.

The presented method enabled segmentation of GFAP-positive cells. While the thin histological slices and the elongated nature of astrocytes prevented direct counting of astrocyte numbers, the GFAP+ fraction of a tile can be considered as a measure of astrogliosis. False-positive signals originating from blood cells, for example, erythrocytes, can be mitigated in future experiments, for example, by co-staining with markers such as Glycophorin A or using antibodies with different wavelengths.

In conclusion, a method for GFAP segmentation in whole-brain slices was presented, enabling spatial investigation of astrocyte response to localized radiation-induced brain damage at the microscopic level. The GFAP expression in mice that developed BBB disruption after focused irradiation linearly increased in the high-dose region with the prescription dose. The combination of the presented method with further markers (e.g. microglia) and inclusion of different timepoints and larger sample size will allow comprehensive understanding of the cellular response and regional differences of focal radiation-induced late effects, enabling the development of new therapeutic and preventive strategies.

## Data Availability

The data that support the findings of this study can be shared on request to the corresponding author.
